# Association between iron status, iron deficiency anaemia, and severe early childhood caries: a case–control study

**DOI:** 10.1186/1471-2431-13-22

**Published:** 2013-02-07

**Authors:** Robert J Schroth, Jeremy Levi, Eleonore Kliewer, James Friel, Michael EK Moffatt

**Affiliations:** 1 Department of Preventive Dental Science, Faculty of Dentistry, University of Manitoba, 507-715 McDermot Avenue, Winnipeg, MB R3E 3P4, Canada; 2 Department of Pediatrics & Child Health, Faculty of Medicine, University of Manitoba, 507-715 McDermot Avenue, Winnipeg, MB R3E 3P4, Canada; 3 The Manitoba Institute of Child Health, 507 – 715 McDermot Avenue, Winnipeg, MB, R3E 3P4, Canada; 4 Winnipeg Regional Health Authority, 4th Floor – 650 Main Street, Winnipeg, MB, R3B 1E2, Canada

**Keywords:** Early childhood caries, Iron, Iron deficiency, Anaemia, Preschool child

## Abstract

**Background:**

Severe tooth decay is known to affect the health and well-being of young children. However, little is known about the influence of Severe Early Childhood Caries (S-ECC) on childhood nutritional status. The purpose of this study was to contrast ferritin and haemoglobin levels between preschoolers with S-ECC and caries-free controls.

**Methods:**

Children were recruited as part of a larger case–control study examining differences in nutritional status between those with and without S-ECC. Preschoolers with S-ECC were recruited on the day of their dental surgery, while caries-free controls were recruited from the community. Parents completed a questionnaire and the child underwent venipuncture. The study was approved by the University’s Health Research Ethics Board. Statistics included descriptive, bivariate and logistic regression analyses. A p value ≤ .05 was significant. A total of 266 children were recruited; 144 with S-ECC and 122 caries-free.

**Results:**

The mean age was 40.8 ± 14.1 months. The mean ferritin concentration for all children was 29.6 ± 17.9 μg/L while the mean haemoglobin level was 115.1 ± 10.1 g/L. Children with S-ECC were significantly more likely to have low ferritin (p=.033) and low haemoglobin levels (p>.001). Logistic regression analyses revealed that children with S-ECC were nearly twice as likely to have low ferritin levels and were over six times more likely to have iron deficiency anaemia than caries-free controls.

**Conclusions:**

Children with S-ECC appear to be at significantly greater odds of having low ferritin status compared with caries-free children and also appear to have significantly lower haemoglobin levels than the caries-free control group. Children with S-ECC also appear to be at significantly greater odds for iron deficiency anaemia than cavity-free children.

## Background

Despite the epidemic nature of both dental caries and iron deficiency worldwide, there has been little research as to whether an association exists between the two conditions. Severe Early Childhood Caries (S-ECC) is defined as the presence of any smooth surface caries for children under the age of 3 and the presence of one or more smooth surface lesions in any primary maxillary anterior teeth for those 3 to 5 years of age (or a dmft score of ≥ 4 (age 3), ≥ 5 (age 4), or ≥ 6 (age 5)) [1]. The implications of S-ECC can extend beyond the oral cavity as it can affect childhood health and well-being [2].

Early Childhood Caries (ECC) is a broad definition of any decay in the primary dentition of children > 72 months of age. It can pose a significant threat to the oral health of young children and its prevalence in North America has increased over the past two decades [3]. Recent Canadian reports suggest that the prevalence of ECC may be approximately 40–50%, but can be significantly higher in economically disadvantaged, First Nations, and immigrant populations [4-8].

While dental surgery is the most common day surgical procedure at most Canadian pediatric hospitals [9,10], we know little about the influence of S-ECC on childhood nutritional status. Many with S-ECC are believed to be malnourished, anaemic, underweight, and have altered somatic growth patterns [11-16]. It is plausible that those with S-ECC are also deficient in important vitamins and nutrients. While several studies suggest that children with S-ECC are underweight, new evidence also suggests that these individuals fall at either extreme of the normal distribution for body mass index (BMI) [13].

According to the World Health Organization (WHO), iron deficiency is the most common and widespread form of nutritional deficiency worldwide, estimating that millions are iron deficient and upwards of two billion anaemic [17]. The WHO estimates the prevalence of anaemia to be 7.6% in Canada and 3.1% in the USA [17]. However, not all cases of anaemia are due to iron deficiency [17].

Though often used synonymously, there are distinctions between being iron deficient, anaemic, and having iron deficiency anaemia. Anaemia is a condition in which afflicted individuals have too few red blood cells or haemoglobin functioning at a suboptimal level. Anaemia is indicative of poor nutrition and health and is generally diagnosed by abnormally low haemoglobin concentrations [17]. However, haemoglobin is also sometimes used as a proxy measure for iron deficiency [17]. Individuals classified as iron deficient have insufficient iron, and are unable to maintain the normal physiological function of tissues which rely on this micronutrient [18]. Iron deficiency can affect a child’s physical and mental development, and is generally identified by low haemoglobin and/or ferritin levels [18]. Iron deficiency can occur without anaemia if its duration has been short or not critical enough to cause haemoglobin levels to fall below set thresholds [18]. Iron depletion, in contrast, occurs when the level of iron stored within the body is negligible, yet tissues are still able to maintain physiologic function [18]. Further, iron deficiency anaemia refers to the scenario in which an iron deficient individual’s lack of iron becomes so severe that they may be considered anaemic [17]. Estimates suggest that nearly half of anaemia cases are due to such instances of iron deficiency [17]. Chronic infections are also known to lower haemoglobin levels, which may also contribute to anaemia [17].

While research relating S-ECC to overall nutritional status is sparse, one small Canadian study explored the possibility of an association between S-ECC and iron levels [14]. This group reported that nearly 80% of children having dental surgery to treat S-ECC had low ferritin levels and 28% had low haemoglobin concentrations [14]. Further, 6% were categorized as iron deficient while 11% had iron deficiency anaemia [14].

The purpose of this study was to contrast iron levels between children with S-ECC and caries-free controls as part of a larger study investigating the relationship between S-ECC and childhood nutritional status.

## Methods

Children were recruited as part of a larger case–control study exploring differences in iron, vitamin D, calcium, and other metabolites between those with and without S-ECC. This study was approved by the University of Manitoba’s Health Research Ethics Board, the Misericordia Health Centre (MHC), and the Health Sciences Centre (HSC). All parents or caregivers of participating children provided informed consent. From October 2009 to August 2011, healthy children > 72 months of age with S-ECC were primarily recruited from the MHC in Winnipeg, Canada on the day of their dental surgery. All had severe tooth decay involving multiple primary teeth necessitating rehabilitative dental surgery under general anaesthetic (GA). Age-matched cavity-free controls were recruited from the community. Controls first underwent a dental assessment, without radiographs, by one of the study team members (RJS) to ensure they were caries-free (dmft = 0).

Parents and caregivers completed an interviewed questionnaire that asked a series of questions pertaining to each child’s nutritional habits, use of supplements, physical and oral health, oral hygiene and dental habits, socioeconomic status (e.g. household income), and family demographics.

Serum samples were analyzed for ferritin, haemoglobin, and Mean Corpuscular Volume (MCV), as they are key biochemical indicators of iron status [18]. Haemoglobin was selected as an indicator of iron status as the protein relies on iron to function. It is also a recognized indicator of anaemia [18]. Ferritin is another protein used as an indicator of iron levels and serves as a container for iron storage within the body. The blood iron level is directly correlated with the blood ferritin level, making it an appropriate measure of blood iron. MCV was also assessed as low levels may serve as an indicator of microcytic anaemia.

Samples were collected from the S-ECC group while in the operating room by the attending anaesthesiologist. For the caries-free children, a topical anesthetic (EMLA) was applied to the anticubital fossa one hour prior to minimize discomfort from the venipuncture. Serum samples were immediately transported to the Clinical Chemistry Laboratory at HSC for analysis. Normal laboratory reference values were adopted to determine whether each child had adequate or low concentrations of ferritin (> 20 μg/L), haemoglobin (> 115 g/L), and MCV (> 75 fL). Reference ranges for ferritin, haemoglobin, and MCV were 20–140 μg/L, 115–135 g/L, and 75–78 fL, respectively. Further, the reported thresholds utilized by other research groups were also applied to the study data (for both ferritin and haemoglobin) [14]. Participants were considered to be iron deficient if they had both abnormal haemoglobin and ferritin concentrations [14]. In addition, iron deficiency anaemia (having two out of three abnormal blood tests for haemoglobin, ferritin, and/or MCV) was also determined [14]. The WHO’s age–appropriate ranges for acceptable haemoglobin were also applied as a further indicator of anaemia. Those having haemoglobin levels below the listed cut–off points (110 g/L for children under 5 and 115 g/L for children under 12) were considered anaemic [17].

Clinical and questionnaire data were entered into an Excel (Microsoft Office) database and analyzed using Number Cruncher Statistical Software (NCSS) version 7.0 (Kaysville, Utah). Analyses included descriptive statistics (frequencies and means ± Standard Deviations (SD)) and bivariate analyses including Chi-Square analysis and t-Tests. Logistic regression for extremely low ferritin (i.e. iron depletion) and iron deficiency anaemia were conducted to assess the relationship with S-ECC while controlling for potential confounders such as income and multivitamin use. A p value ≤ 0.05 was statistically significant.

## Results

A total of 266 children were recruited; 144 with S-ECC and 122 caries-free. Laboratory reports were unavailable for four children (three with S-ECC and one control). The mean age of all participating children was 40.8 ± 14.1 months. There were no significant differences in mean age (p=0.14) or sex (p=0.37) between the groups. Table 1 presents descriptive information about participants and their caregivers. 

**Table 1:** Characteristics of participating Children

**Variable**	**Overall value**	**Study group**		**P Value**
		**Caries-Free**	**S-ECC**	
Mean Age (months) ± S.D.*	40.8 ± 14.1	39.4 ± 16.3	42.0 ± 11.9	.14
Sex†				
Male	136	66 (48.5%)	70 (51.5%)	.37
Female	130	56 (43.1%)	74 (56.9%)	
Mean Height (cm) ± S.D.*	98.4 ± 9.6	97.3 ± 11.1	99.3 ± 8.1	.094
Mean Weight (Kg) ± S.D.*	16.4 ± 3.7	15.8 ± 4.0	17.0 ± 3.3	**.010**
Multivitamin Use†				
No	123 (46.2%)	55 (44.7%)	68 (55.3%)	.73
Yes	143 (53.8%)	67 (46.9%)	76 (53.1%)	
Caregiver Status†				
Mother	247 (92.9%)	115 (46.6%)	132 (53.4%)	
Father	15 (5.6%)	6 (40.0%)	9 (60.0%)	.62
Other	4 (1.5%)	1 (25.0%)	3 (75.0%)	
Yearly Household Income†				
≤ $28,000	119	34 (28.6%)	85 (71.4%)	**>.0001**
> $28,000	134	87 (64.9%)	47 (35.1%)	

*T-test.

†Chi-Square.

**Figure 1:**
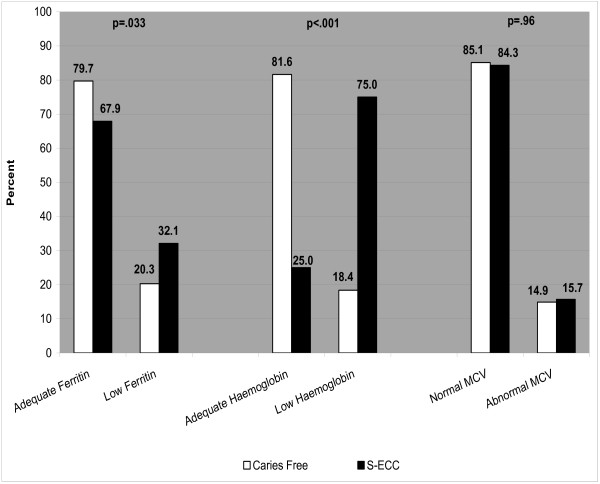
Distribution of Ferritin, Haemoglobin, and MCV between Caries-Free and S-ECC Groups.

Overall, the mean ferritin concentration for all participating children was 29.6 ± 17.9 μg/L (range 3–132) while the mean haemoglobin level was 115.1 ± 10.1 g/L (range 76–139). *T*- test analysis revealed that ferritin levels did not differ significantly between the groups (29.1 ± 18.4 μg/L S-ECC vs. 30.2 ± 17.4 caries-free, p=.62). However, children with S-ECC had significantly lower mean haemoglobin levels than controls (109.8 ± 8.7 vs. 121.7 ± 7.6, p>.001). Children were categorized into low/normal groupings for ferritin and haemoglobin based on their clinical chemistry results. Chi-square analysis revealed that children with S-ECC were significantly more likely to have low ferritin concentrations (defined as > 20 μg/L) than cavity-free controls (p=.033) (Figure 1). 

Likewise, children with S-ECC were more likely to have low haemoglobin levels (p>.0001). There was no significant difference in MCV levels between those with and without S-ECC (78.3 ± 5.3 vs. 78.5 ± 3.8, p=.74) and no difference in the proportion of children with abnormal MCV levels (56.4% vs. 43.6%, p=.86).

**Figure 2:**
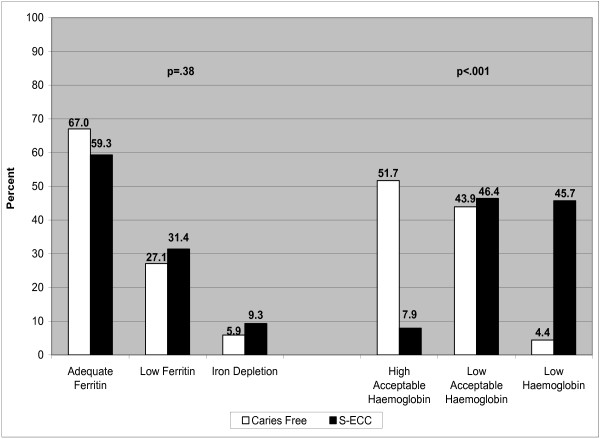
**Distribution of Ferritin and Haemoglobin by other Published Thresholds **[14].

When previously published thresholds [14] were applied, the majority (62.8%) had ferritin concentrations in the acceptable range (22.1–400 μg/L), 29.5% had low levels (10.1–22 μg/L), while 7.7% were iron deplete (> 10 μg/L). Although 57.9% of the children who had low iron levels and 65.0% of the children who were iron deplete belonged to the S-ECC group, there was no significant difference between the groups (p=.38) (Figure 2). 

Adopting previously published thresholds for haemoglobin revealed a significant difference between groups (p>.001); with a higher proportion in the S-ECC group having low haemoglobin concentrations.

There was no difference in the reported use of multivitamins between the two groups (p=.73). Based upon reports of children’s dietary habits, ferritin levels were not found to be associated with the frequency of dietary intake of iron-rich foods (e.g. red meats (beef, pork, lamb), poultry, eggs, green vegetables including spinach, lentils, beans, nuts, and dried fruits) (data not shown). However, children who took multivitamins had significantly higher ferritin levels than those who did not (31.7 ± 20.2 μg/L vs. 27.0 ± 14.4, p=.03).

Several children met the conditions for being “iron deficient” as they had both unacceptably low levels of ferritin and haemoglobin. A total of 37 (14.6%) fit this category. Children with S-ECC were significantly overrepresented in the iron deficient category as they accounted for 91.9% of cases while only 8.1% (n=3) belonged to the caries-free group (p>.0001 Fisher’s Exact Test).

Children were considered to have iron deficiency anaemia if they had at least two of three abnormal tests for ferritin, haemoglobin, and/or MCV. A total of 48 children (18.9%) were ultimately determined to be in a state of iron deficiency anaemia. Overall 83.3% (n=40/48) of children who were found to have iron deficient anaemia had S-ECC (p>.0001). Additionally, when using the WHO’s cut-offs for anaemia (based on haemoglobin), it was found that 29.5% of the entire study sample were anaemic. Of this group of children, 92% (n=69/75) were from the S-ECC group compared with only 8% (n=6) in the caries-free cohort (p>.001).

Two logistic regression models were developed to control for potential confounders (Table 2). One model used severely low ferritin status (i.e. iron depletion) as the dependent variable and controlled for study grouping, multivitamin use, and household income. Results indicated that children with S-ECC were at nearly twice the odds of being classified as having low ferritin. The other regression model was for iron deficiency anaemia and revealed that children with S-ECC were over six times the odds of having iron deficiency anaemia. 

**Table 2:** Logistic regression for low Ferritin and Iron deficiency Anaemia

	**Variable**	**Regression coefficient (b)**	**Standard error (b)**	**Adjusted odds ratio**	**± 95% Confidence interval**	**P Value**
Severely Low Ferritin (i.e. Iron Depletion)	S-ECC	0.64	0.32	1.89	0.0047, 1.27	**.048**
	(Reference: Yes)					
	Multivitamin Use	−0.26	0.30	0.77	−0.85, 0.33	.39
	(Reference: Yes)					
	Yearly Household Income	−0.038	0.32	0.96	−0.67, 0.59	.91
	(Reference: >$28,000)					
Iron Deficiency Anaemia	S-ECC	1.88	0.45	6.58	1.01, 2.76	**<.0001**
	(Reference: Yes)					
	Multivitamin Use	−0.69	0.36	0.50	−1.39, 0.014	.055
	(Reference: Yes)					
	Yearly Household Income	0.43	0.38	1.54	−0.31, 1.18	.25
	(Reference: > $28,000)					

## Discussion

Until very recently there has been minimal research in the area of nutritional iron status and caries. This study provided an opportunity to explore this relationship by comparing the ferritin and haemoglobin levels between children undergoing rehabilitative dental surgery for S-ECC and cavity-free children recruited from the community.

While there was no statistically significant difference observed between the two groups with respect to average iron concentrations, there was a significant difference in the number of children exhibiting low ferritin levels. In fact, our study reveals that those undergoing dental surgery were significantly more likely to be classified as having low ferritin (p=.033). This is largely congruent with findings from another Canadian team that identified 80% of their dental surgery cohort as having unacceptable ferritin levels and 28% having low haemoglobin status [14]. Our present study displayed a similar trend, as 70.5% of participants had unacceptable ferritin levels. In fact, the adjusted odds of children with S-ECC having low ferritin was nearly double that of cavity-free children. More recently, another group has reported a similar significant relationship between rampant caries during childhood and low ferritin status [19]. They reported that children with rampant caries had significantly lower levels of ferritin, haemoglobin, and iron than a group of caries-free controls [19]. Both studies as well as our own study suggest that there is in fact a relationship between S-ECC and overall iron levels.

Overall, we found that children with S-ECC had significantly lower haemoglobin levels than the caries free controls. This included differences in mean haemoglobin concentrations and groupings based on existing laboratory thresholds. Meanwhile, when previously published definitions of iron deficiency and iron deficiency anaemia [14] were applied to the study data, we found that the S-ECC group was overrepresented in both conditions. Compared to our Canadian colleagues, we observed a higher prevalence of both iron deficiency (14.6% vs. 6%) as well as iron deficiency anaemia (18.9% vs. 11%) [14]. As a whole, the observations made by the present study show agreement with their findings, though the higher prevalence of iron deficiency and iron deficiency anaemia (even with the inclusion of a control group) suggests that children from these regions of Manitoba with S-ECC may be at an elevated risk.

When the WHO’s groupings for anaemia were applied, 29.5% of our entire study sample were found to be anaemic. Meanwhile, the proportion of Canadian preschool children estimated to be anaemic is only 7.6%. This statistic, however, relied on a slightly different threshold value (with only those falling below 110 g/L being considered anaemic) which, when applied to the study data, indicated that 27.6% of our entire sample was anaemic.

The two other recent reports on iron status and severe caries are novel, but have some limitations [14,19]. One did not include a comparison group of cavity-free children and only observed those with rampant decay undergoing rehabilitative surgery [14]. The other recruited their children with severe caries based upon a definition of having a microcytic anaemia due to an underlying iron deficiency [19]. As such, it is expected that these children would naturally display lower levels of iron. Despite this shortcoming, their work provides evidence that the relationship between iron levels and S-ECC is salient. Interestingly, this group also observed significant improvements in ferritin and haemoglobin levels after rehabilitative dental surgery [19]. While the specific nature of this relationship is currently unknown, there are several plausible explanations as to why the iron levels of a child are associated with the presence of S-ECC. One hypothesis is that the low haemoglobin levels often observed in S–ECC children may be attributed to the body’s inflammatory response, which may accompany rampant forms of dental caries (especially those involving pulpitis or abscesses). Inflammation associated with S–ECC may trigger a series of events which ultimately leads to the production of cytokines, which may, in turn, inhibit erythropoiesis and thus reduce the level of haemoglobin in the blood [20] (and therefore the level of iron). The reduction of haemoglobin levels is a common occurrence in many chronic diseases and, if severe enough, may lead to “anaemia of chronic disease”. S-ECC may be one such chronic disease. It is also recognized that the pain experienced by children with S–ECC may lead to altered eating habits [2]. These eating habits may lead to nutritional deficiencies such as low iron levels. Additionally, differences in nutritional status between caries-free children and those with S-ECC may also be shaped by household economics. Limited funds may restrict a family’s ability to purchase nutritious foods. Low socioeconomic status is known to be associated with increased risk for anaemia [21]. We attempted to control for the influence of household finances in our logistic regression models and still found that after controlling for yearly household income and multivitamin use that children with S-ECC were at increased risk of having a nutritional deficiency.

The implications of a relationship between iron and S-ECC have the potential to be far-reaching, as a child’s iron status has been demonstrated to have a significant impact on health. For instance, learning and memory deficits, decreased fine motor skills, and increased anxiety may all be observed in children suffering from iron deficiency [22]. The ability to recognize early warning signs of low iron levels (such as S-ECC) may allow patients to receive the necessary interventions before the longstanding effects of iron deficiency are able to take root. Perhaps pre-operative assessments for children requiring dental surgery to treat S-ECC should include evaluation of iron and haemoglobin levels.

This study was not without its limitations. The study design was cross-sectional and does not allow for the determination of true cause and effect. While our groups were essentially matched by sex and age, we were not able to match by socioeconomics. Since S-ECC is influenced by the social determinants of health, more children in this group were from lower income households. It was also very challenging to find cavity-free peers from similar neighbourhoods and backgrounds to participate. For obvious reasons there are some differences between children with and without S-ECC that are impossible to control for as they are key determinants (e.g. household income, parental education, etc.). While we did not measure caries rates in the S-ECC group, all had multiple cavitated carious lesions requiring treatment under GA. While informative and applicable to the general population of S-ECC children, the results from this study are not necessarily transferrable to the general population of Manitoban preschoolers. Despite these challenges, the sample size provided sufficient statistical power to assess whether associations were present.

## Conclusion

Based upon the findings from this study we conclude that:

1. Children with S-ECC appear to be at significantly greater odds of having low ferritin status compared with caries-free children.

2. Children with S-ECC appear to have significantly lower haemoglobin levels when compared with caries-free controls.

3. Children with S-ECC appear to be at significantly greater odds for iron deficiency and iron deficiency anaemia than cavity-free children.

Primary care providers and dentists should be aware of this oral-systemic relationship. Future studies could assess whether or not pediatric dental rehabilitative surgery to treat S-ECC helps to restore iron and haemoglobin levels to normal ranges.

## Abbreviations

dmft: decayed, missing, filled teeth; ECC: Early Childhood Caries; MCV: Mean Corpuscular Volume; MHC: Misericordia Health Centre; HSC: Health Sciences Centre; SD: Standard Deviation; S-ECC: Severe Early Childhood Caries; WHO: World Health Organization

## Competing interests

The authors declare that they have no competing interests.

## Authors’ contributions

RJS: Conception and design, acquisition of data, analysis and interpretation of data, drafting of article, revising article critically for important intellectual content, and final approval of version to be published. JL: Acquisition of data, analysis and interpretation of data, drafting of article, revising article critically for important intellectual content, and final approval of version to be published. EK: Acquisition of data, revising article critically for important intellectual content, and final approval of version to be published. JF: Analysis and interpretation of data, revising article critically for important intellectual content, and final approval of version to be published. MEKM: Conception and design, analysis and interpretation of data, revising article critically for important intellectual content, and final approval of version to be published.

## Pre-publication history

The pre-publication history for this paper can be accessed here:

http://www.biomedcentral.com/1471-2431/13/22/prepub
